# Antioxidant, Antiproliferative and Anti-Enzymatic Capacities, Nutritional Analysis and UHPLC-PDA-MS Characterization of Ungurahui Palm Fruits (*Oenocarpus bataua* Mart) from the Peruvian Amazon

**DOI:** 10.3390/antiox11081598

**Published:** 2022-08-18

**Authors:** Gabriel Vargas-Arana, Claudia Merino-Zegarra, Ángel Martín Rodríguez del-Castillo, Cristina Quispe, Ezequiel Viveros-Valdez, Mario J. Simirgiotis

**Affiliations:** 1 Laboratorio de Química de Productos Naturales, Instituto de Investigaciones de la Amazonía Peruana, Avenue Abelardo Quiñones, Iquitos 16001, Peru; 2 Facultad de Industrias Alimentarias, Universidad Nacional de la Amazonía Peruana, Iquitos 16001, Peru; 3 Facultad de Ciencias de la Salud, Universidad Arturo Prat, Iquique 1110939, Chile; 4 Facultad de Ciencias Biológicas, Universidad Autónoma de Nuevo León, San Nicolas de los Garza 66450, Nuevo León, Mexico; 5 Facultad de Ciencias, Instituto de Farmacia, Universidad Austral de Chile, Valdivia 5090000, Chile

**Keywords:** antioxidant activity, UHPLC-DAD, cytotoxic, nutritional values, ESI-MS, phenolics, cholinesterase, *Oenocarpus*

## Abstract

Ungurahui, or Patawa, fruits are a popular fruit and medicinal food used in the Amazon. Here, we have studied nine natural populations of ungurahui from the Peruvian Amazon regarding their nutritional and biological activities, including metal composition, proximal analyses, cytotoxic, antioxidant and cholinesterase inhibition activities. Twenty-four compounds have been detected in these Peruvian natural populations by UHPLC-MS, including nine phenolic acids (peaks 1–6, 8, 9 and 11), four C-glycosyl flavonoids (peaks 12, 16, 17 and 18), two flavonols (peaks 7 and 10), one flavanol (peak 15), three anthocyanins (peaks 13, 14 and 22) and five resveratrol derivatives (peaks 19–21, 23 and 24). Sample 9, Tunaants, showed the highest DPPH clearing capacity regarding the content of Trolox equivalents (2208.79 μmol Trolox/g), but an ORAC test of the sample collected in San Lorenzo showed the highest clearing activity (1222.28 μmol Trolox/g) and the sample collected in Allpahuayo Mishana showed the most powerful ABTS (1803.72 μmol Trolox/g). The sample from Jenaro Herrera was the most powerful in AChe inhibition (IC_50_ 2.05 ± 0.03 μg/mL), followed by the sample from Contamana (IC_50_ 2.43 ± 0.12 μg/mL). In BChE inhibition, the sample from Palestina was the most active (4.42 ± 0.06 μg/mL), followed by samples from Tunaants and San Lorenzo. The differences among bioactivities can be related to the different growing conditions of the populations of ungurahui. The palm tree fruit proved to be a good source of natural antioxidants and dietary fatty acids, and their consumption represents an alternative for the prevention of neurodegenerative or related non-chronic transmittable diseases.

## 1. Introduction

**Figure 1:**
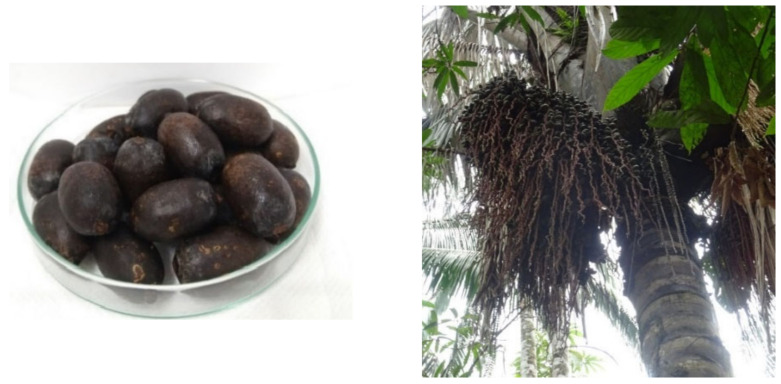
Fruits (**right**) of ungurahui palm trees (**left**).

In the Amazonian tropical area, palm tree species are an omnipresent component of the flora. Palms from the Amazon have been used since the earliest times as a source of materials, food and natural medicines by local people [1]. Local people use palm trees in everyday life, and their fruits are highly appreciated for their unique flavor and nutritional properties. In Amazonian Peru, several palm species are used for the preparation of widely appreciated traditional drinks or ice-creams. *Oenocarpus bataua* Mart, more commonly called ungurahui or patawa, is an Amazonian palm tree fruit widespread in the neotropical rainforests. It grows wild and has a darkly colored oleaginous edible fruit (Figure 1) with a pleasant and nutritious flavor that is consumed daily, especially in the Peruvian Amazon. This palm tree is from the Areceae tribe and belongs to the same subtribe (Euterpeinae) as assai and bacaba, which are very popular fruits, especially in Brazil [2].

Known phytochemical information about *O. bataua* Mart mostly pertains to its fatty acid and tocopherol composition [2,3]. However, a few studies about the chemistry of the fruits [4] and leaves [5] have been reported, but no natural populations from Peru have been compared to date. Moreover, a study regarding three distinct genetic clusters of *Onocarpus bataua* in Peru was carried out by Escobar et al. [6], but currently, there are no studies about the comparison of the chemical composition and biological activities between individuals of these genetic clusters, which can serve as a basis for the identification of the best populations that can be used for cultivation and/or repopulation of this species.

**Figure 2:**
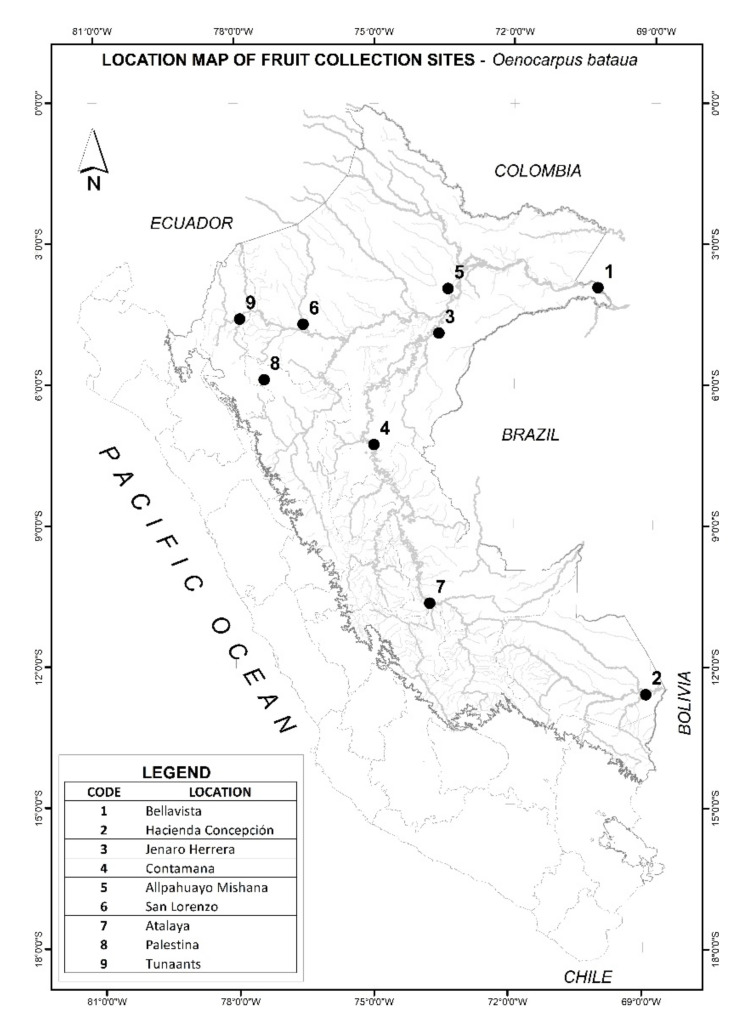
Collection sites of ungurahui fruits *Oenocarpus bataua* natural populations.

The present study examines the chemical (full HPLC-MS of phenolic composition, fatty acid content, minerals, nutritional values) and biological activities (antioxidant and antiproliferative, plus anticholinesterase activities) of ungurahui (*Oenocarpus bataua* Mart) fruits growing in natural populations of nine different locations belonging to three genetic clusters in the Peruvian Amazon (Figure 2).

## 2. Materials and Methods

### 2.1. Chemicals

HPLC methanol and formic acid (for mass spectrometry) were obtained from J.T. Baker (Phillipsburg, NJ, USA). Ultra-pure water (<5 μg/L TOC) was obtained from a purification system (Arium 126 61316-RO), plus an Arium 611 UV unit (Sartorius, Goettingen, Germany). Commercial Folin–Ciocalteu reagent, ferric chloride hexahydrate, 2,2-diphenyl-1-picrylhydrazyl (DPPH), 2,4,6-tris(2-pyridyl)-s-triazine, Trolox, dimethyl sulfoxide (DMSO), Amberlite^®^ resin (XAD-4), tyrosinase (EC1.14.18.1), acetylcholinesterase (AChE, EC 3.1.1.7), butyrylcholinesterase (BChE, EC 3.1.1.8), phosphate buffer, L-DOPA, kojic acid, trichloroacetic acid (Merck, Darmstadt, Germany), fetal calf serum (FCS, Gibco, Grand Island, NY, USA), L-glutamine (Merck, Darmstadt, Germany), sodium persulfate sodium carbonate, ferrous sulfate, sodium acetate, sodium sulfate anhydrous and absolute ethanol were obtained from Sigma Aldrich Chem. Co. (Sigma, St. Louis, MO, USA) and HPLC standards (gallic acid, (±)-α-tocopherol, quercetin, chlorogenic acid, quercetin 3-O-glucoside, vicenin 2, orientin, vitexin and isovitexin purity 95% by HPLC) were obtained from Merck (Lima, Perú), Extrasynthèse (Genay, France), or Phytolab (Vestenbergsgreuth, Germany).

### 2.2. Plant Material and Sample Treatment

The study was carried out with *Oenocarpus bataua* fruits of nine natural populations located in different localities selected from Peruvian Amazon (Table 1, Figure 1 and Figure 2 and Figure S1 in Supplementary Material). Collection points were selected with the intention of covering localities within the three distinct genetic clusters identified in Peru for *Onocarpus bataua* [6]. In this sense, mature, ripe fruits, free of fungal infection, injury from insects and physical damage were selected from those collection places and transported to the laboratory. The fruits were pulped manually with a knife, removing the seeds. The whole fruit was then lyophilized at −55 °C and 0.021 mbar for 72 h (Alpha 1-2 LDplus, Christ, Germany). Finally, the dried fruit material was reduced to a fine powder using a blade mill (Grindomix GM 200, Retsch, Haan, Germany), wrapped in plastic bags and stored in a freezer at −20 °C until use. The enriched phenolic extracts for biological activities and HPLC analysis were prepared by extracting each of the lyophilized and defatted fruits (500 mg, defatting with 10 mL hexane three times) with 10 mL of ethanol with 1% formic acid using ultrasound-assisted extraction (ELMA ultrasonic bath, GmbH, Germany) at 600 W and 35 kHz frequency for 10 min. After extraction, the supernatants were combined and centrifuged for 30 min at 9000 g, and then filtered through Whatman paper number 1, and concentrated in vacuum at 40 °C to give the final extracts (yields around 10%), which were stored at −20 °C. Collection sites are georeferenced in Table 1.

### 2.3. UHPLC-PDA-MS Instrument

HPLC-MS analysis was performed as described by [7] with some modifications. Briefly, a Scientific Ultimate 3000 UHPLC apparatus connected to a Thermo Q exactive plus machine was used (Thermo Fischer Inc., Bremen, Germany). For the analysis the extract (1 mg) was dissolved in 1 mL of methanol, filtered (PTFE filter), and 10 μL were injected in the instrument, with all specifications set as follows: Parameters Full MS scan: Maximum IT: 80 ms, AGC target: 5 × 10^6^ Resolution: 35,000 Range: 100–1500 *m*/*z*, Microscans: 1, Parameters MS2 Maximum IT: 100 ms AGC target: 1 × 10^6^, Carrier gas: N_2_ (Sheath gas flow rate: 48, Sweep gas flow rate: 2), Resolution: 17,500, Ionization source parameters: ESI (positive and negative) spray volt: 3.5/2.5 KV, Capillary temperature: 260 °C, Gas heater temp: 280/280 °C, S-lens RF level: 100.

### 2.4. LC Parameters and MS Parameters

Fruit material from the nine *Oenocarpus bataua* populations selected from Peruvian Amazon was lyophilized, defatted with hexane and extracted with ethanol, and these extracts (Section 2.2) were injected into the UHPLC system (10 μL at 1 mg/mL) and analyzed for phenolic components according to our previous standard protocol [7], using a C-18 Luna© column (Omega C-18 100 A, Phenomenex 150 mm × 2.1 mm × 1.6 μm) operated at 25 °C. The detection lines were 254, 280, 330 and 354 nm, and PDA at 200–800 nm was recorded for peak identification. Mobile phases were as follows (A): 1% aqueous formic solution and (B) acetonitrile with 1% formic acid. The gradient program in time (min), and percentage B) was as follows: (0.00 min, 5% B); (1.500 min, 15 B); (1.5 min, 5%B); (35.00 min, 95% B); (36.00, 95% B); (38.00 min, 5% B); 15 min wait time before each injection. The flow rate was 0.300 mL/min, and the injection volume was 10 μL. Standards and the fruit extracts dissolved in methanol were kept at 10 °C during storage in the autosampler. The compounds were characterized by accurate high-resolution molecular weight determination (HRAM) and using typical mass fragmentation patterns of classical phenolic compounds in Ungurahui and UV-vis spectra, which were obtained by photodiode array (PDA) detector, plus literature reviews of full mass spectral data.

### 2.5. Antioxidant Activity Assays

#### 2.5.1. DPPH Test

The DPPH• radical was assayed by the decolorization method [8]. DPPH• radical (3.9 mL, 100 μM dissolved in 80% methanol), was added 0.1 mL of the dissolved extract (at 2 mg/mL in 80% methanol), previously filtered on a disk filter (0.45 μm), the mixture was left in the dark for 30 min at 25 °C with stirring. The absorbance was then taken at 517 nm using a UV-visible Cary60 spectrophotometer (Agilent, Santa Clara, CA, USA). The concentration of DPPH• was generated from a calibration curve by linear regression. The results were expressed in TEAC, or antioxidant activity equivalent to Trolox (μmol Trolox/g of extract). The synthetic reference antioxidant Trolox, at a concentration of 5–30 μM in 80% methanol solution, was tested under the same conditions. Three experiments were performed individually for the test and standard solution and the values were recorded as the mean ± SD (Y = 2.1871X + 2.6219; R^2^ = 0.9996).

#### 2.5.2. ABTS Bleaching Capacity

The ABTS assay was performed by bleaching the cationic radical ABTS•+ as described by Re et al. [9]. For the preparation of the radical ABTS•+ 2.5 mL of the 7 mM ABTS solution, it was mixed with 2.5 mL of 2.45 μM sodium persulfate for 12 h in the dark at 4 °C. Then, the resulting solution was diluted with absolute ethanol until an initial absorbance of approximately 0.70 ± 0.02 was obtained at 734 nm. The reaction started with the addition of 1500 μL of ABTS•+ solution to 500 μL of the extract (2 mg/mL) in a cuvette kept at 30 °C. It was stirred vigorously and left to react for 7 min, then the absorbance was read at 734 nm using a Cary60 UV-visible spectrophotometer (Agilent, Santa Clara, CA, USA). The results are expressed in TEAC (μmol Trolox/g of extract). The calibration curve for TEAC was constructed using different concentrations of Trolox (4–14 μM, Y = 3.9763X + 19.808; R^2^ = 0.996) in PBS buffer solution under the same conditions. Three experiments were performed individually for the test and the values were recorded as the mean ± SD.

#### 2.5.3. ORAC Assay

A previously stated method was employed [10]. AAPH was used as peroxyl generator. In this assay, Trolox calibration solutions, 40 μL of sample, plus blank were mixed in triplicate. The fluorescence of fluorescein disodium (FL) of each cycle was measured by using a Synergy HTX microplate reader (Biotek, Winooski, VT, USA). The plate reader parameters were cycle time, 210 s; orbital shaking (4 mm shake width), position delay, 0.3 s; injection speed, 420 μL/s, cycle number, 35; shaking mode, 8 s before each cycle. Calculations were performed by a quadratic regression equation between the Trolox or sample concentration and net area under the FL decay curve. Data are expressed as micromoles of Trolox equivalents (TE) per gram of sample (μmol of TE g^−1^). Three experiments were performed in triplicate for each measurement and the values are recorded as the mean ± SD, while the area under curve (AUC) was calculated as follows:

AUC = 0:5 + f4/f3 + f5/f3 + f6/f3 + ⋯ + f32/f4 + f33/f4) × CT

where f3 = fluorescence reading at cycle 3; fn = fluorescence reading at cycle n; CT, cycle time in minutes. (Y = 0.1451X + 1.4962; R^2^ = 0.9721). (Figure S2 Supplementary Material).

### 2.6. Polyphenol (Folin-Ciocalteu Test) Content

The content of phenols was obtained by a colorimetric method described by Velioglu et al. [11] with some modifications. Briefly, 100 μL of the extract (at 2 mg/mL in 80% methanol) was mixed with 750 μL of the Folin-Ciocalteu reagent (diluted in a 1/10 proportion of Milli-Q water). After 5 min in the dark, 750 μL of sodium bicarbonate (60 g/L) was added to the mixture. The tubes were kept in the dark for 90 min at 30 °C, then the absorbance was obtained at 725 nm using a Cary60 UV-visible spectrophotometer (Agilent, Santa Clara, CA, USA). Gallic acid (10–100 μg, Y = 0.0076X − 0.0182; R^2^ = 0.9998) was used for the preparation of the standard curve. The content regarding phenolic compounds was then expressed as mg gallic acid per gram of extract. Three experiments were performed in triplicate for each measurement and the values were recorded as the mean ± SD.

### 2.7. Tocopherol Contents (Vitamin E)

Tocopherol analysis was performed by HPLC according to described by Rezaire et al. [4], with some modifications. Lyophilized fruit (3 g) were placed in a flask with ascorbic acid (1 g), methanol (150 mL) and potassium hydroxide (50 mL; at 50 g/100 mL) under reflux and nitrogen atmosphere for 40 min at 80 °C. After saponification and cooling of the flask with water, the extraction of tocopherols was performed with dichloromethane (10 mL, three times each, for 10 min with sonication). The apparatus consisted of an HPLC chromatograph (Hitachi LaCrhom Elite^®^, Tokyo, Japan) equipped with a vacuum degasser a quaternary pump, and a Diode-Array Detector (DAD). Twenty microliters of crude extract dissolved in methanol (1 mg/mL) were injected into a column (RP-18E, 250 × 4.6 nm, 5 μm). The mobile phase was water/methanol (3/97 *v*:*v*), and flow rate was 1.5 mL/min at 30 °C. Tocopherol detection was carried out by using a spectrofluorometer (excitation wavelength: 295 mM; emission wavelength: 330 nm). Three experiments were performed in triplicate for each measurement and the values were recorded as the mean ± SD. Concentrations were calculated using the areas of peaks. Calibration curves were obtained using commercial α-tocopherol as standard. LOD and LOQ for α-tocopherol were 1.75 and 5,20 μg/mL, respectively, (Y = 171.61X − 3.6903; R^2^ = 0.998). Results were obtained at mg of α-tocopherol equivalent/100 g of dry matter.

### 2.8. Antiproliferation Activity Assay

The cytotoxicity activity and antiproliferative effects of extracts were detected with the method as described previously by Viveros-Valdez et al. [12]. HeLa (ATCC^®^ CCL-2), MCF-7 (ATCC^®^ HTB-22) and HT-29 (ATCC^®^ HTB-38) cancer cell lines were used. The cells were seeded in 25 cm^2^ tissue culture flasks in Dulbecco’s modified eagle medium (DMEM) and added 10% fetal bovine serum (FBS), with a pH adjusted to 7.2. A mixture of antibiotics composed of streptomycin-penicillin (10,000 μg/mL: 10,000 IU/mL; 1 mL of mixture/1L of medium) was added. Cells were incubated at constant humidity at 37 °C under a 5% CO_2_/95% air atmosphere. Cells were harvested by trypsinization, and an assessment made of their density via haemocytometer (bright line, Sigma-Aldrich, Saint Louis, MO, USA) and viability by Trypan Blue (4%). The different cancer cell lines were seeded with 5000 cells per well in a 96-well plate. After incubation for 24 h, 100 μL of fruit extracts (62.5, 125, 500, 750 and 1000 μg/mL) were added and incubated for another 24 h. In total, 20 μL of Alamar Blue Invitrogen™ solution (10%) was added to each well. The plate was agitated, and the fluorescence was measured after 4 h in a fluorometer FLx800 (BioTek Instruments, Winooski, VT, USA, 535 nm excitation and emission at 595 nm wavelength). Culture medium without any extract or compound was used as the zero control (no dead cells). Paclitaxel (Taxol), an antiproliferative and cytotoxic agent, was used as the positive control. Results are presented as mean SD values. Each measurement was performed at least in triplicate. The IC_50_ values were calculated by probit analysis [13].

### 2.9. Determination of Cholinesterase Inhibition

Inhibition of cholinesterase activity was performed according to the Ellman method, as reported previously [10], by using a Synergy HTX microplate reader (Biotek, Winooski, VT, USA). The samples (2 mg/mL) were prepared by mixing the tris-HCl buffer (50 mM, pH 8.0), the reactant 5-dithio-bis (2-nitrobenzoic acid) (DTNB) at pH 8.0 and the enzyme solution (AChE or BChE). The reaction started by simply adding acetyl-thiocholine iodide (ATCI) or butyryl-thiocholine chloride (BTCl). The absorbance of the extract was measured at 405 nm after 30 min at 37 °C. Three experiments were performed in triplicate in each test and the values were recorded as the mean ± SD. The results were expressed as IC_50_ (μg/mL, the final concentration of the plate ranged from 0.05 to 25 μg/mL). Galantamine was used as a positive control.

### 2.10. Determination of Proximal Composition

AOAC procedures were used in all determinations [14,15]. Moisture content was determined by using an oven to dry the sample (fresh fruit) to a constant weight, the crude protein content obtained by the Kjeldahl method (N × 6.25), using a Kjelmaster k375 (Büchi Labortechnik AG, Flawil, Switzerland) apparatus and the fiber content by gravimetric method after acidic hydrolysis of the samples, while the total lipid obtained by means of a Soxhlet apparatus (Sigma-Aldrich, Saint Louis, MO, USA) using petroleum ether as solvent, the ash content taken by a muffle furnace incineration at 550 ± 15 °C. Total carbohydrates were calculated as the following difference: 100 − (g water + g protein + g fiber + g fat + g ash). Results were expressed in g per 100 g fresh weight (g/100 g fw).

### 2.11. Mineral Analysis

For the mineral analysis, the fresh pulps were dried to ash at 550 °C [15,16]. The ash was boiled in each case with 10 mL of 20% hydrochloric acid and then filtered into a 100 mL standard flask and made up to 100 mL with deionized water. Levels of minerals potassium (K), sodium (Na), calcium (Ca), magnesium (Mg), zinc (Zn), copper (Cu), manganese (Mn) and iron (Fe) were determined from the resulting solution using atomic absorption spectroscopy (Varian AA240, Belrose, Australia), previously calibrated with standard solutions containing known amounts of the minerals being determined with analytical grade reagents. The following two types of flames were used: air-acetylene and nitrous oxide-acetylene, the latter only for calcium analysis. Monometallic hollow cathode lamps were used for each element analyzed. All analyses were performed in triplicate.

### 2.12. Physicochemical Analysis and Fatty Acid Profile

Physicochemical analysis (acidity index, peroxide index, saponification index, iodine index, unsaponifiable matter, refractive index and density), of the oils extracted from the lyophilized pulps with petroleum ether at cold, were carried out according to the AOCS recommended methodology [17]. The fatty acid profile (Figure S3 Supplementary Material) was obtained by gas chromatography of fatty acid methyl esters (FAME). The oils were converted to their corresponding methyl esters, which were prepared by saponification and esterification with potassium hydroxide in methanol (2 M). Fatty acid methyl esters were extracted with hexane and processed on a Varian 450-GC gas chromatograph (Walnut Creek, CA, USA). The chromatograph was equipped with a VF-WAXms, 60 m × 0.25 mm ID, 0.25 μm capillary column, CP9207, flame ionization detector and Varian CP-8400 autoinjector. Helium was used as carrier gas. The temperature program used was as follows: 3 min at 130 °C; gradual heating at 220 °C for 9 min; 35 min at 220 °C, cooling to 130 °C and 130 °C for 5 min. The detector temperature was 280 °C and the injector temperature was 245 °C [18]. Esterified fatty acids were identified and quantified by comparison with the known retention times of previously injected standards. For C16, C18, C18:1, C18:2 fatty acids, LODs were: 2.2, 2.5, 2.3 and 3.4, and LOQs were: 25, 26, 28 and 54 ng, respectively.

### 2.13. Statistical Analysis

The statistical analysis was performed using the software package originPro 9.1 (Originlab Corporation, Northampton, MA, USA). The determination was performed at least three times for each sample solution. Significant differences between means by the Tukey comparison (*p* values < 0.05 were regarded as significant).

## 3. Results and Discussion

### 3.1. Nutritional and Physicochemical Properties of Ungurahui Fruits

Table 2 shows the proximal composition of ungurahui fruits from nine natural populations, such as the humidity, ashes, protein, lipids, carbohydrates and fiber, while Table 3 shows the mineral contents (macro and micronutrients), and Table 4 shows the physicochemical properties of crude oils from ungurahui fruits. The results of composition showed that the caloric value and nutritional support of this fruit are similar to that shown for other palm species [1], but with a high crude protein (from 3.07 to 17.58 percent), fiber (from 11.58 to 22 percent) and carbohydrate content (from 12.10 to 27.50 percent). The edible portion of the fruits was analyzed for mineral content (Ca, Na, Mg, K, Cu, Mn, Zn and Fe). The ungurahui fruits of all-natural populations were generally high in manganese (from 34.47 to 65.29 mg Mn/100 g edible portion), low in sodium (from 0.63 to 2.78, except for sample 4 with 17.39 mg Na/100 g edible portion) and moderate in magnesium (from 34.41 to 50.93 mg Mg/100 g edible portion) and copper (from 0.58 to 1.06 mg Cu/100 g edible portion). The values found for manganese and copper are in agreement with the recommended daily amounts in adults, Cu (1 mg) and Mn (2 mg) [19]. Regarding fatty acids in total, some samples showed more fatty acid content than that previously reported from Brazil (14.4 g/100 g) [2], and total proteins in these Peruvian natural populations were higher than that reported by Darnet et al. (4.9 g/100 g) [2], Table 4, making those Peruvian population fruits more healthy foods than their Brazilian counterparts. The physicochemical properties of crude oils and fatty acid identification profiles of crude oils from ungurahui fruits were different from those reported by Serra et al. [17]. For instance, the acid values were lower than the Brazilian sample by Serra et al. (7.52 mg KOH/g) and the peroxide value (20.85 meqO_2_/kg) and iodine index were higher (73.18 g I/100 g) [17]. Six fatty acids were identified in this study. Oleic acid was the majority in all natural populations, followed by palmitic acid (Table 5). These results are consistent with previous studies carried out by other authors [2,3,20]. However, sample 6, San Lorenzo, showed the highest oleic acid percent (83.35 ± 0.61%). The proportion of unsaturated fatty acids in ungurahui oil is comparable to olive oil, thus, this oil should be considered within our diet for its good nutritional value. Differences observed for the different populations of different parameters measured can be due to the different environments (precipitation, soil, and temperature).

### 3.2. Metabolite Profiling Using UHPLC-ESI-MS/MS

**Figure 3:**
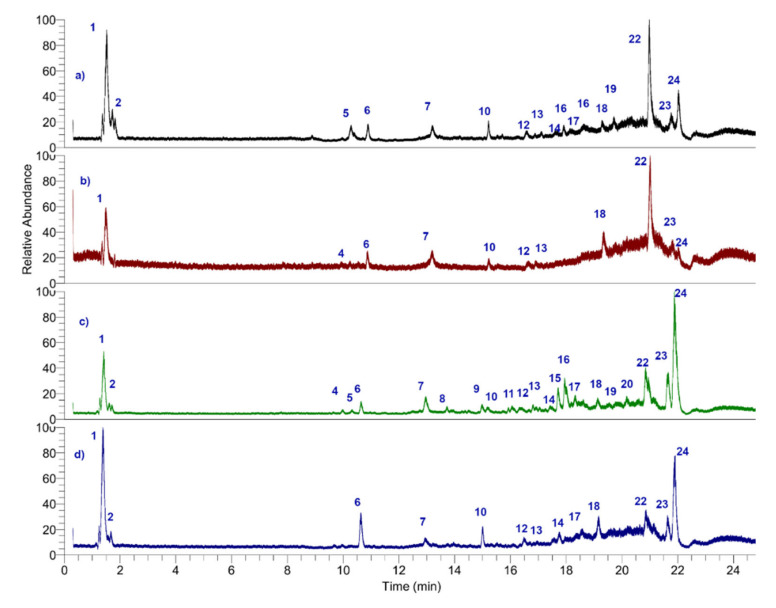

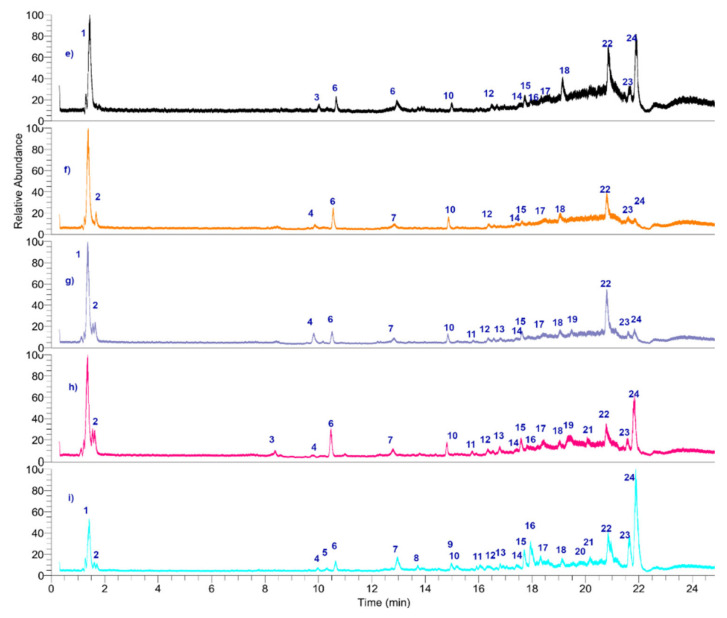
UHPLC-PDA-ESI-OT-MS/MS chromatograms (TIC, total ion current) of ungurahui fruits 1–9 from natural populations.

**Figure 4:**
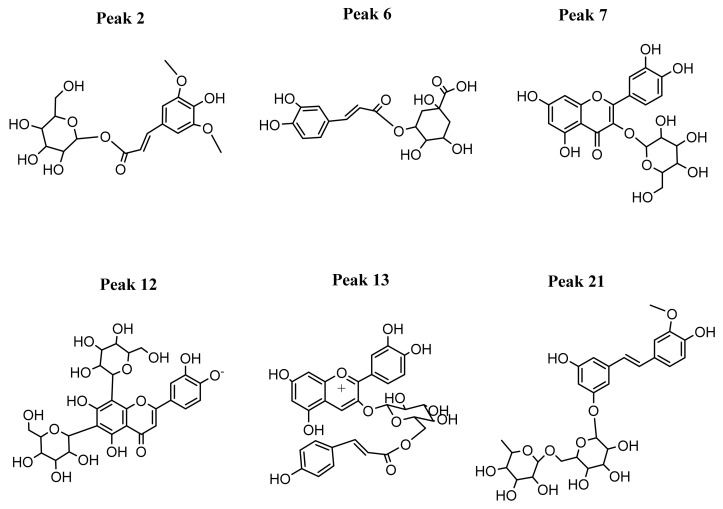
Structures of some representative phenolic compounds detected in ungurahui fruits: spermidine, peak 2, 1-*O*-Sinapoyl-glucoside, peak 6, chlorogenic acid, peak 7, quercetin 3-*O*-glucoside, peak 12, vicenin 2, peak 13, cyanidin 3-*O*-(6-*O*-*p*-coumaroyl) glucose and peak 21, methoxyresveratrol rutinose.

Metabolite profiling was carried out with all fruits with the corresponding defatted extract by HPLC MS, for identification purposes, and fatty acids and carotenoids were quantified. Figure 3 shows the full total ion current UHPLC-mass chromatograms of ungurahui fruits from natural populations 1–9, and Table 6 shows the comprehensive MS analysis and tentative identification of metabolites detected in the fruits from natural populations. Figure 4 shows the structures of some representative phenolic compounds. Below are the detailed analyses. Figure S4, Supplementary Material, shows some examples of UHPLC Q-orbitrap MS spectra. The MS daughter ions are diagnostic ions for the identification of the parent compounds in each group case.

#### 3.2.1. Anthocyanins

Three known anthocyanins were identified in the fruits, (peaks 13, 14 and 22) with molecular ions in positive mode at *m*/*z* 595.1451, 433.1126 and 595.1659 (C_30_H_27_O_13_, C_21_H_20_O_10_ and C_27_H_31_O_15_) and showing diagnostic MS^2^ ions at *m*/*z* 303 (MS^3^ ion at *m*/*z* 257), 287 (MS^3^ ions at *m*/*z* 213, 147), 317(MS^3^ ion at *m*/*z* 302), 301 (MS^3^ ion at *m*/*z* 286) and 331(MS^3^ ion at *m*/*z* 299, 179) respectively, corresponding to cyanidin 3-*O*-(6-*O*-*p*-coumaroyl) glucose, pelargonidin 3-*O*-glucoside and cyanidin-3-O-rutinoside (λ max: 275–341sh-512), ref. [21] respectively. The identity was verified by co-elution with standard anthocyanins and literature data.

#### 3.2.2. Flavonoids

Peak 7 and peak 10 were identified as the related compounds quercetin 3-O-glucoside [22] and isorhamnetin-3-*O*-glucoside, respectively (Table 6). Peak 15, with UV max at 280 nm and a pseudomolecular ion at *m*/*z*: 433.1138, was identified as naringenin-5-*O*-glucoside (C_21_H_22_O_10_). Peak 16 with an M-H- ion at *m*/*z*: 447.09148 was identified as the luteolin-6-C-glucoside, isoorientin (C_21_H_19_O_11_^−^, Uv max: 270 nm) and peak 18 as the related apigenin-6-C-glucoside, isovitexin (MS: 431.09837) [23], both showing typical diagnostic losses of 60, 90 and 120 Daltons. In the same manner, peak 12 with an M-H- ion at *m*/*z*: 609.14609 was identified as the apigenin di C-glucoside, vicenin 2 (C_27_H_29_O_16_) [24].

#### 3.2.3. Resveratrol Derivatives

Peak 19 with an M-H- ion at *m*/*z*: 581.1870 was identified as methoxyresveratrol diglucose (C_27_H_33_O_14_), ref. [4]. Similarly, peak 20 was identified as methoxyresveratrol diglucose (C_27_H_33_O_14_), peak 21 as methoxyresveratrol rutinose and peak 23 as resveratrol diglucose and peak 24 as a hydroxylated derivative of the latter [4]. Daughter resveratrol ions are diagnostic fragments for identification (Table 6).

#### 3.2.4. Phenolic Acids

Peak 1 was identified as quinic acid, peak 2 was identified as 1-*O*-sinapoyl-glucoside (C_17_H_22_O_10_) [25] and peak 4 as syringic acid glucoside (C_15_H_19_O_10_). Peaks 5 and 6 showing adduct 2 M-H-ions at *m*/*z*: 707.18420 and father ion at around *m*/*z*: 353.08826, and daughter ions at around *m*/*z*: 191.05603 (quinic moiety) were identified as isomers of caffeoyl quinic acid (chlorogenic acid), ref. [26] peak 8 as the related 3,5-dicaffeoylquinic acid (C_25_H_23_O_1__2_^−^) and peak 9 as 3-caffeoyl-5-feruloylquinic acid (C_26_H_25_O_12_^−^), ref. [10] peak 11 as methyl chlorogenate (C_17_H_20_O_9_), while finally, peak 3 with an M-H- ion at *m*/*z*: 515.1407 was identified as 4-O-(3′-*O*-glucopyranosyl)-caffeoyl quinic acid (C_22_H_28_O_14_). Daughter quinic acid and feruloyl acid moiety ions are diagnostic fragments for the identification (Table 6).

### 3.3. Antioxidant Activity, Total Polyphenol and Tocopherol Contents, Antiproliferative and Cholinesterase Activities

In this study, the antioxidant capacities of the nine natural populations were assessed by the trapping of ABTS, DPPH and ORAC by fluorometry and expressed as μmol Trolox/g dry extract (Table 7). The ORAC test is more sensitive than the others and is based on the fluorescence method measuring peroxyl radicals was developed by DeLange and Glaze in 1989. This is the most reliable test used in the food and fruit industry. In addition, the Folin and Ciocalteu method for total phenolic contents was assessed and correlated with the antioxidant capacities. Trapping of ABTS and DPPH radicals showed similar values in the different natural populations and was correlated with phenolic contents for the dry and defatted fruits. Sample 9, Tunaants, showed the highest DPPH clearing capacity in terms of the content of Trolox equivalents (2208.79 μmol Trolox/g), but interestingly, in the ORAC test, sample 6, San Lorenzo, showed the highest clearing activity (1222.28 μmol Trolox/g) and in ABTS, the most powerful was sample 5, Allpahuayo Mishana (1803.72 μmol Trolox/g). These antioxidant activity values are similar to those reported by Rezaire et al. [4] for one sample of ungurahui and close to that of the popular fruit assai. Differences in the correlation between the antioxidant assays (Table 7) could be due to the different nature of the tests since the oxygen radical absorption capacity (ORAC) test is based on the transfer of a hydrogen atom and is different from the total phenolic Folin–Ciocalteu’ test, which is based on the transfer of one electron, and the tests involving the transfer of both an electron and a hydrogen atom, which include the 2,2′-Azinobis-(3-ethylbenzothiazoline-6-sulfonic acid (ABTS) test and the [2,2-di(4-tert-octylphenyl)-1-picrylhydrazyl] (DPPH) test. However, a good correlation was found between total phenolic and the DPPH (r = 0.8615, *p* < 0.001) and ABTS (r = 0.7718, *p* < 0.0001) antioxidant assays. The values obtained for all samples on tocopherol contents (from 6.46 to 8.19 mg α-tocopherol/100 g dry fruit) are consistent with those found in the literature [2,3,4]. The cholinesterase activities of the natural populations of ungurahui are presented in Table 8. Sample 3 was the most powerful in AChe inhibition (IC_50_ 2.05 ± 0.03 μg/mL), followed by sample 4 (IC_50_ 2.43 ± 0.12 μg/mL), and in BChE inhibition, sample 8 was the most active (4.42 ± 0.06 μg/mL), followed by samples 9 and 6 (Table 8). Phenolic compounds are shown to be active against cholinesterases. Indeed, some polar phenolic compounds from South American plants proved to be active against these enzymes. For instance, the polar flavonoid isoastilbin [7] showed good inhibition activity explained by docking into cholinesterase sites AChE: 4.68 ± 0.03 (51.70% at 2.2 μM) and BChE: 8.51 ± 0.03 (50.10% at 2.2 μM) enzymes. Other phenolics isolated, such as glycosylated apigenin and kaempferol, showed binding energies of 9.76 and −2.04 kcal/mol for *Tc*AChE and −5.93 and −8.92 for *h*BChE, respectively, in the active sites of these enzymes [10]. These reports show the importance of phenolics in fruits that could be suitable for use in the prevention of neurodegenerative diseases. Different biological activities found can be related to small differences in phenolic composition, fatty acids and tocopherols due to differences in growing conditions of the different ungurahui locations and also differences in the values already published for other Amazonian locations.

The antiproliferative activity of the different extracts was examined by the method of cell culture using the human estrogen-dependent breast cancer MCF-7, colon cancer Ht-29 and cervix HeLa tumor cell lines. IC_50_ values (concentration inhibiting the growth of cell culture by 50%) were obtained from the concentration of the tested extract, using graphics of cell viability relationships (expressed as a percentage in relation to control). The IC_50_ values of samples are shown in Table 9. As stated by the National Cancer Institute (NCI, USA), a crude extract can be considered active when IC_50_ ≤ 20 (μg/mL). It is interesting to note the almost exclusive effect that the analyzed extracts showed against the growth of hormone-dependent MCF-7 breast cancer cells, which allows us to suppose that the inhibitory effect is more related to the structure of certain flavonoids (as apigenin and luteolin) with its anti-estrogenic capacity [27], that with the antioxidant and antienzimatic (AChE and BChE) capacity, likewise it has already been shown that some flavonoids and resveratrols since several resveratrols derive molecules could be detected. In this sense, the phytoalexin resveratrol, found in grapes and other fruits and derived products, is an anticancer compound and is also an anti-inflammatory, antimutagen and antioxidant. It was proven to induce phase II drug-metabolizing enzymes and inhibit cyclooxygenase and hydroperoxidase [28]. On the other hand, it is important to consider that these fruits have been safely consumed since pre-Hispanic times by Amazonian populations, which could explain the poor or no cytotoxic/antiproliferative activity that they showed against Ht-29 and HeLa cell lines at the doses analyzed.

## 4. Conclusions

In this study, 24 compounds were detected in natural populations of ungurahui fruits from the Peruvian Amazon. Of those, nine were phenolic acids, (peaks 1–6, 8, 9 and 11), four C-glycosyl flavonoids (peaks 12, 16, 17 and 18), two flavonols (peaks 7 and 10), one flavanol (peak 15), three anthocyanins (peaks 13, 14 and 22) and five resveratrol derivatives (peaks 19–21, 23 and 24). Most of the samples showed excellent antioxidant activity, while some samples showed good activity against inhibition of cholinesterase enzymes, and some samples were lightly antiproliferative against the MCF-7 breast cancer cell line. Our study showed that the chemistry plus the bioactivity results obtained with some different natural populations of ungurahui fruits, which can be considered outstanding, opens the door for the potential use of this plant to manage chronic diseases. The cultivation and/or repopulation of this palm tree, selecting the outstanding natural populations as a seedbed, provides this common fruit with the ability to be a rich source of bioactive substances that can boost its consumption, not only as a food product but also as a natural medicine. Ungurahui’s chemistry and bioactivity give this species great potential for bio business, improving the quality of life of the Amazonian people who live from the forest resources.

## Figures and Tables

**Table 1:** Georeferencing of collection sites of ungurahui fruits.

Sample	Location	District	Province	Region	Latitude	Longitude
1	Bellavista	Santa Rosa de Yavarí	Mariscal Ramón Castilla	Loreto	03°56′13.34″ S	70°13′28.66″ W
2	Hacienda Concepción	Tambopata	Tambopata	Madre de Dios	12°36′34.27″ S	69°04′49.12″ W
3	Jenaro Herrera	Jenaro Herrera	Requena	Loreto	04°55′11.63″ S	73°36′48.57″ W
4	Contamana	Contamana	Ucayali	Loreto	07°18′38.93″ S	75°00′10.76″ W
5	Allpahuayo Mishana	San Juan Bautista	Maynas	Loreto	03°57′48.52″ S	73°25′09.40″ W
6	San Lorenzo	Barranca	Datem del Marañón	Loreto	04°43′55.49″ S	76°31′02.79″ W
7	Atalaya	Atalaya	Atalaya	Ucayali	10°42′26.79″ S	73°47′40.29″ W
8	Palestina	Nueva Cajamarca	Rioja	San Martín	05°55′0.00″ S	77°21′15.19″ W
9	Tunaants	Nieva	Condorcanqui	Amazonas	04°36′55.36″ S	77°52′14.99″ W

**Table 2:** Proximal composition of ungurahui fruit pulp expressed in g/100 g in fresh weight.

Sample	Humidity	Ashes	Total Lipids	Crude Protein	Crude Fiber	Carbohydrates
1	37.42 ± 0.38 ^a^	1.05 ± 0.02 ^a^	16.19 ± 0.58 ^a^	6.69 ± 0.01 ^a^	22.00 ± 0.47 ^a^	16.65
2	28.50 ± 0.92 ^b^	1.41 ± 0.02 ^b^	22.37 ± 0.19 ^b^	13.98 ± 0.14 ^b^	18.78 ± 0.68 ^b^	14.96
3	32.50 ± 0.50 ^c^	1.19 ± 0.01 ^c^	23.01 ± 0.35 ^b^	11.66 ± 0.99 ^c^	19.54 ± 0.93 ^b^	12.10
4	21.65 ± 0.41 ^d^	1.93 ± 0.03 ^d^	25.98 ± 0.97 ^c^	11.64 ± 0.07 ^c^	15.68 ± 0.25 ^c^	23.12
5	35.33 ± 0.52 ^e^	1.81 ± 0.02 ^e^	18.14 ± 0.68 ^d^	17.58 ± 0.12 ^d^	11.58 ± 0.06 ^d^	15.56
6	44.95 ± 0.71 ^f^	1.29 ± 0.03 ^f^	13.88 ± 0.12 ^e^	5.82 ± 0.34 ^e^	14.31 ± 0.34 ^ce^	19.75
7	47.51 ± 0.22 ^g^	1.30 ± 0.03 ^f^	15.87 ± 0.16 ^a^	3.19 ± 0.33 ^f^	12.13 ± 0.29 ^d^	20.00
8	26.91 ± 0.51 ^h^	1.85 ± 0.02 ^e^	22.36 ± 0.79 ^b^	7.56 ± 0.22 ^g^	13.82 ± 0.61 ^e^	27.50
9	39.85 ± 0.31 ^i^	1.45 ± 0.01 ^b^	13.60 ± 0.10 ^e^	3.07 ± 0.17 ^f^	15.54 ± 0.50 ^ce^	26.49

Values marked with the same letter in the same column are not statistically different (*p* < 0.05). Samples: 1: Bellavista, 2: Hacienda Concepción, 3: Jenaro Herrera, 4: Contamana, 5: Allpahuayo Mishana, 6: San Lorenzo, 7: Atalaya, 8: Palestina, 9: Tunaants.

**Table 3:** Mineral content (mg/100 g fresh pulp) of ungurahui fruits.

Sample	K	Na	Ca	Mg	Zn	Cu	Mn	Fe
1	3.50 ± 0.11 ^a^	1.44 ± 0.06 ^a^	49.41 ± 1.51 ^a^	44.46 ± 1.25 ^a^	1.00 ± 0.02 ^a^	1.06 ± 0.02 ^a^	3.63 ± 0.07 ^a^	0.56 ± 0.02 ^a^
2	8.08 ± 0.21 ^b^	1.07 ± 0.05 ^b^	15.32 ± 0.58 ^b^	42.90 ± 1.01 ^a^	0.79 ± 0.03 ^b^	0.93 ± 0.02 ^b^	3.58 ± 0.15 ^a^	1.00 ± 0.01 ^b^
3	4.32 ± 0.20 ^c^	1.15 ± 0.06 ^ab^	12.70 ± 0.39 ^bc^	50.63 ± 0.77 ^b^	1.28 ± 0.07 ^c^	0.68 ± 0.02 ^c^	3.04 ± 0.10 ^b^	0.68 ± 0.02 ^c^
4	8.31 ± 0.22 ^b^	17.39 ± 0.36 ^c^	88.14 ± 2.40 ^d^	65.29 ± 2.11 ^c^	0.86 ± 0.04 ^b^	0.86 ± 0.03 ^d^	4.23 ± 0.21 ^c^	1.18 ± 0.04 ^d^
5	4.07 ± 0.17 ^cd^	1.75 ± 0.06 ^d^	41.23 ± 1.05 ^e^	50.93 ± 2.06 ^b^	0.52 ± 0.02 ^d^	0.58 ± 0.01 ^e^	5.82 ± 0.12 ^d^	0.71 ± 0.07 ^c^
6	3.80 ± 0.18 ^ad^	1.00 ± 0.02 ^b^	6.88 ± 0.25 ^f^	34.41 ± 1.17 ^d^	0.61 ± 0.03 ^de^	0.72 ± 0.02 ^c^	4.51 ± 0.12 ^c^	0.77 ± 0.05 ^c^
7	4.46 ± 0.12 ^c^	0.63 ± 0.03 ^e^	69.99 ± 1.50 ^g^	48.12 ± 0.73 ^ab^	0.74 ± 0.03 ^bf^	0.95 ± 0.01 ^b^	10.60 ± 0.41 ^e^	0.58 ± 0.02 ^a^
8	6.72 ± 0.08 ^f^	2.78 ± 0.06 ^f^	10.96 ± 0.30 ^c^	41.11 ± 1.76 ^a^	0.66 ± 0.03 ^ef^	0.80 ± 0.01 ^f^	3.36 ± 0.09 ^ab^	0.73 ± 0.03 ^c^
9	4.57 ± 0.10 ^c^	1.38 ± 0.04 ^ab^	15.46 ± 0.67 ^b^	45.23 ± 1.13 ^a^	0.60 ± 0.03 ^de^	0.90 ± 0.03 ^bd^	3.13 ± 0.06 ^ab^	0.66 ± 0.03 ^ac^

Values marked with the same letter in the same column are not statistically different (*p* < 0.05). Sample: 1: Bellavista, 2: Hacienda Concepción, 3: Jenaro Herrera, 4: Contamana, 5: Allpahuayo Mishana, 6: San Lorenzo, 7: Atalaya, 8: Palestina, 9: Tunaants.

**Table 4:** Physicochemical properties of crude oils from ungurahui fruits.

Sample	Acidity Index (mgKOH/g)	Peroxide Index (meqO_2_/kg)	Saponification Index (mgKOH/g)	Iodine Index (gI/100 g)	Unsaponificable Matter (%)	Refractive Index (25 °C)	Density (g/cm^3^)
1	3.59 ± 0.03 ^a^	7.05 ± 0.23 ^a^	187.06 ± 0.60 ^a^	117.44 ± 0.20 ^a^	0.61 ± 0.01 ^a^	1.465 ± 0.00	0.91 ± 0.00
2	4.14 ± 0.06 ^b^	9.44 ± 0.23 ^b^	180.90 ± 0.50 ^b^	122.12 ± 0.20 ^b^	0.86 ± 0.03 ^b^	1.464 ± 0.00	0.91 ± 0.00
3	4.66 ± 0.02 ^c^	8.91 ± 0.23 ^bc^	180.34 ± 1.06 ^b^	112.36 ± 0.34 ^c^	0.75 ± 0.03 ^c^	1.459 ± 0.00	0.92 ± 0.00
4	3.52 ± 0.16 ^a^	7.72 ± 0.23 ^d^	171.61 ± 0.32 ^c^	129.43 ± 0.22 ^d^	0.51 ± 0.01 ^d^	1.452 ± 0.00	0.91 ± 0.00
5	1.90 ± 0.08 ^d^	7.32 ± 0.23 ^ad^	177.82 ± 0.22 ^d^	122.54 ± 0.20 ^be^	0.89 ± 0.01 ^b^	1.462 ± 0.00	0.91 ± 0.00
6	1.39 ± 0.03 ^e^	7.50 ± 0.22 ^ad^	182.43 ± 1.36 ^b^	120.92 ± 0.26 ^f^	0.88 ± 0.02 ^b^	1.454 ± 0.00	0.91 ± 0.00
7	1.39 ± 0.03 ^e^	7.85 ± 0.23 ^d^	179.59 ± 0.47 ^bd^	116.86 ± 0.33 ^a^	0.87 ± 0.04 ^b^	1.432 ± 0.00	0.91 ± 0.00
8	2.16 ± 0.07 ^f^	8.65 ± 0.23 ^c^	171.42 ± 0.29 ^c^	123.31 ± 0.16 ^e^	0.98 ± 0.04 ^e^	1.461 ± 0.00	0.90 ± 0.00
9	1.32 ± 0.05 ^e^	7.85 ± 0.23 ^d^	179.91 ± 0.54 ^b^	120.21 ± 0.80 ^f^	0.85 ± 0.04 ^b^	1.448 ± 0.00	0.91 ± 0.00

Values marked with the same letter in the same column are not statistically different (*p* < 0.05). Sample: 1: Bellavista, 2: Hacienda Concepción, 3: Jenaro Herrera, 4: Contamana, 5: Allpahuayo Mishana, 6: San Lorenzo, 7: Atalaya, 8: Palestina, 9: Tunaants.

**Table 5:** Fatty acids profile of crude oils from ungurahui fruits.

Sample	Fatty Acid								
	C16:0 (Palmitic)	C16:1 (Palmitoleic)	C18:0 (Stearic)	C18:1 (Oleic)	C18:2 (Linoleic)	C18:3 (Linolenic)	Saturated FAs	Mono-UFAs	Poly-UFAs
1	13.22 ± 0.64 ^a^	0.38 ± 0.01 ^a^	2.75 ± 0.03 ^a^	80.87 ± 0.61 ^a^	2.10 ± 0.05 ^a^	0.68 ± 0.01 ^a^	15.97	81.25	2.78
2	12.17 ± 0.31 ^b^	0.27 ± 0.01 ^b^	4.96 ± 0.03 ^b^	78.01 ± 0.61 ^b^	3.87 ± 0.05 ^b^	0.72 ± 0.01 ^b^	17.13	78.28	4.59
3	11.93 ± 0.28 ^b^	0.21 ± 0.01 ^c^	3.44 ± 0.03 ^c^	81.73 ± 0.61 ^ac^	2.03 ± 0.05 ^a^	0.66 ± 0.01 ^c^	15.37	81.94	2.69
4	11.87 ± 0.24 ^b^	0.47 ± 0.01 ^d^	3.99 ± 0.03 ^d^	79.34 ± 0.61 ^d^	3.66 ± 0.05 ^c^	0.67 ± 0.01 ^d^	15.86	79.81	4.33
5	12.53 ± 0.34 ^ab^	0.27 ± 0.00 ^b^	3.92 ± 0.03 ^d^	78.41 ± 0.61 ^bd^	4.12 ± 0.05 ^d^	0.75 ± 0.00 ^b^	16.45	78.68	4.87
6	11.60 ± 0.26 ^b^	0.39 ± 0.01 ^a^	1.47 ± 0.03 ^e^	83.35 ± 0.61 ^e^	2.70 ± 0.05 ^e^	0.49 ± 0.01 ^a^	13.07	83.74	3.19
7	12.43 ± 0.01 ^ab^	0.27 ± 0.00 ^b^	5.07 ± 0.03 ^b^	77.42 ± 0.61 ^b^	4.07 ± 0.05 ^bd^	0.74 ± 0.00 ^b^	17.50	77.69	4.81
8	12.54 ± 0.14 ^ab^	0.49 ± 0.01 ^d^	1.55 ± 0.03 ^e^	82.29 ± 0.61 ^cf^	2.39 ± 0.05 ^f^	0.74 ± 0.01 ^b^	14.09	82.78	3.13
9	11.43 ± 0.15 ^b^	0.36 ± 0.01 ^a^	2.52 ± 0.03 ^a^	82.76 ± 0.61 ^ef^	2.27 ± 0.05 ^af^	0.66 ± 0.01 ^a^	13.95	83.12	2.93

Values marked with the same letter in the same column are not statistically different (*p* < 0.05). Sample: 1: Bellavista, 2: Hacienda Concepción, 3: Jenaro Herrera, 4: Contamana, 5: Allpahuayo Mishana, 6: San Lorenzo, 7: Atalaya, 8: Palestina, 9: Tunaants.

**Table 6:** High resolution UHPLC PDA-Q orbitrap identification of metabolites in fractions of ungurahui fruits of natural populations 1–9.

Peak#	Retention Time (min)	UV Max	Tentative Identification	Molecular Formula	Theoretical Mass (*m*/*z*)	Measured Mass [M − H]^−^ or [M + H]^+^(*m*/*z*)	Accuracy (δ ppm)	MS^n^ Ions (δ ppm)	Sample
1	1.20	208	Quinic acid	C_7_H_11_O_6_^-^	191.0550	191.0557	2.34	135.04477, 85.02844	1–9
2	1.32	329	1-*O*-Sinapoyl-glucoside	C_17_H_22_O_10_	385.1142	385.1147	1.29	247.0612, 223.0611, 205.0504, 190.0269 164.0704, 119.0342	1–9
3	8.21	325	4-*O*-(3′-*O*-Glucopyranosyl)-caffeoyl quinic acid	C_22_H_28_O_14_	515.1401	515.1407	1.16	395.0990, 353.0876, 191.0557, 179.0344 161.0238, 135.0444	8
4	9.25	276, 328	Syringic acid glucoside	C_15_H_19_O_10_	359.0981	359.0983	0.55	197.0453	2, 3, 6–9
5	9.98	213, 287, 326	Neochlorogenic acid	C_17_H_17_O_9_^-^	353.08671	353.08804	3.78	707.18420 (2M-H^-^), 191.05565 (quinic moiety)	3
6	10.12	213, 287, 326	Chlorogenic acid *	C_17_H_17_O_9_^-^	353.08671	353.08826	4.38	707.18463 (2M-H^-^), 191.05603 (quinic moiety)	1–9
7	12.92	255–355	Quercetin 3-*O*-glucoside *	C_21_H_19_O_11_^-^	463.08820	463.08630	4.10	300.02634 271.0251, 255.0301, 178.9982 151.0032 (quercetin aglycone)	1–9
8	13.34	213, 287, 326	3,5-Dicaffeoylquinic acid	C_25_H_23_O_12_^-^	515.11840	515.11932	1.78	353.08795, 135.04449	3, 4
9	15.12	213, 287, 326	3-Caffeoyl-5-feruloylquinic acid	C_26_H_25_O_12_^-^	529.13405	529.13495	3.95	271.06137, 135.04153	3, 9
10	15.32	254, 354	Isorhamnetin-3-*O*-glucoside	C_22_H_21_O_12_^-^	477.10385	477.10159	4.73	314.04166 isorhamnetin aglycone	1–9
11	16.01	325	Methyl chlorogenate	C_17_H_20_O_9_	367.1031	367.1035	1.08	191.0556, 179.0344, 161.0237, 135.0443 93.0335	3, 7
12	16.32	280	Apigenin di C-glucoside vicenin 2	C_27_H_29_O_16_	609.14611	609.14609	0.69	579.17193, 549.1613, 519.1508 521.1306, 491.1195, 473.1453, 457.1193	1–9
13	16.87	520	Cyanidin 3-O-(6-*O*-*p* coumaroyl) glucose	C_30_H_27_O_13_	595.14517	595.14529	0.67	433.1080, 271.0595, 215.0695, 163.0596 127.0389	1–9
14	17.92	520	Pelargonidin 3-*O*-glucoside	C_21_H_20_O_10_	433.1121	433.1126	1.15	311.0556, 269.0460, 163.0031	1, 3–9
15	17.82	280	Naringenin-5-*O*-glucoside	C_21_H_22_O_10_	433.1132	433.1138	1.38	313.0551, 271.0613, 151.0030, 119.0493 93.00335	3, 5–9
16	18.02	265, 335	Luteolin-6-C-glucoside Orientin *	C_21_H_19_O_11_^-^	447.09329	447.09148	−4.04	387.0322, 357.009797, 329.0665, 327.0668, 285.04046, 284.03128	1, 3–9
17	18.23	265,335	Luteolin-8-C-glucoside Isoorientin *	C_21_H_19_O_11_^-^	447.09326	447.09148	−4.04	387.0324, 359.07724, 357.009792, 329.0666, 327.0671, 285.03122	3–9
18	19.12	265,335	Apigenin-6-C-glucoside Isovitexin *	C_21_H_19_O_10_^-^	431.09832	431.09837	−4.04	371.0322, 389.0878, 341.0822, 327.03135, 311.0717, 269.0454	1–9
19	18.53	283–303	Methoxyresveratrol diglucose	C_27_H_33_O_14_	581.1875	581.1870	−0.84	243.0662	3, 6–9
20	20.01	283–303	Methoxyresveratrol diglucose	C_27_H_33_O_14_	581.1875	581.1873	−0.34	243.0662	3, 9
21	20.23	283–303	Methoxyresveratrol rutinose	C_27_H_33_O_13_	565.1926	565.1921	−0.88	243.0662	8, 9
22	21.97	520	Cyanidin-3-*O*-rutinoside	C_27_H_31_O_15_	595.1663	595.1659	−0.67		1–9
23	21.70	283–303	Resveratrol diglucose	C_26_H_31_O_14_	567.1719	567.1712	−1.23	227.0713	1–9
24	22.01	283–303	Hydroxyresveratrol diglucose	C_27_H_33_O_12_	551.1770	551.1765	−0.90	243.0661	1–9

* Identified by spiking experiments with authentic compound. 1–9: ungurahui fruits. 1: Bellavista, 2: Hacienda Concepción, 3: Jenaro Herrera, 4: Contamana, 5: Allpahuayo Mishana, 6: San Lorenzo, 7: Atalaya, 8: Palestina, 9: Tunaants.

**Table 7:** Antioxidant activity (DPPH, ABTS, ORAC), total phenolic and tocopherol contents.

Sample	DPPH (μmol Trolox/g)	ABTS (μmol Trolox/g)	ORAC (μmol Trolox/g)	Total Phenolic (mg AG/g)	Tocopherol Content (mg α-Tocopherol/100 g)
1	1510.94 ± 73.90 ^a^	870.57 ± 16.05 ^a^	869.74 ± 17.75 ^a^	1408.90 ± 27.46 ^a^	6.46 ± 0.12 ^a^
2	1402.18 ± 20.51 ^b^	748.81 ± 14.43 ^b^	521.71 ± 11.87 ^b^	1468.95 ± 25.57 ^ab^	8.19 ± 0.08 ^b^
3	1557.51 ± 17.05 ^a^	892.65 ± 11.55 ^a^	715.81 ± 6.47 ^c^	1428.68 ± 19.74 ^ab^	6.85 ± 0.07 ^c^
4	1356.08 ± 17.93 ^b^	831.51 ± 14.82 ^ac^	687.94 ± 7.65 ^c^	1373.63 ± 16.25 ^a^	6.55 ± 0.04 ^ad^
5	2082.27 ± 41.10 ^c^	1803.72 ± 20.86 ^d^	921.30 ± 3.06 ^d^	2037.70 ± 24.95 ^c^	6.76 ± 0.08 ^cd^
6	962.21 ± 13.46 ^d^	804.64 ± 15.80 ^bc^	1222.28 ± 20.88 ^e^	1003.63 ± 18.31 ^d^	6.63 ± 0.07 ^acd^
7	1727.35 ± 36.07 ^e^	1306.66 ± 31.51 ^e^	1109.19 ± 3.34 ^f^	1475.99 ± 21.00 ^b^	7.23 ± 0.08 ^e^
8	1798.72 ± 24.56 ^e^	1263.57 ± 20.49 ^e^	914.48 ± 17.66 ^g^	1268.19 ± 10.95 ^e^	7.71 ± 0.07 ^f^
9	2208.79 ± 29.54 ^f^	1441.71 ± 26.74 ^f^	997.97 ± 9.01 ^b^	1900.18 ± 29.20 ^f^	6.91 ± 0.07 ^c^

Sample: 1: Bellavista, 2: Hacienda Concepción, 3: Jenaro Herrera, 4: Contamana, 5: Allpahuayo Mishana, 6: San Lorenzo, 7: Atalaya, 8: Palestina, 9: Tunaants. Values marked with the same letter in the same column are not statistically different (*p* < 0.05).

**Table 8:** Antienzimatic (AChE and BChE) activity of ungurahui fruits (IC_50_ ± SD, μg/mL).

Sample	IC_50_ AChE	IC_50_ BChE
1	4.52 ± 0.08 ^a^	7.13 ± 0.10 ^a^
2	8.22 ± 0.06 ^b^	10.40 ± 0.07 ^b^
3	2.05 ± 0.03 ^c^	5.95 ± 0.02 ^c^
4	2.43 ± 0.12 ^d^	22.38 ± 0.04 ^d^
5	2.91 ± 0.04 ^e^	7.02 ± 0.01 ^a^
6	2.82 ± 0.07 ^e^	4.78 ± 0.04 ^e^
7	8.20 ± 0.15 ^b^	11.30 ± 0.04 ^f^
8	6.46 ± 0.04 ^f^	4.42 ± 0.06 ^g^
9	2.52 ± 0.03 ^d^	4.66 ± 0.06 ^e^
Galantamine *	0.26 ± 0.01 ^g^	3.82 ± 0.03 ^h^

Sample: 1: Bellavista, 2: Hacienda Concepción, 3: Jenaro Herrera, 4: Contamana, 5: Allpahuayo Mishana, 6: San Lorenzo, 7: Atalaya, 8: Palestina, 9: Tunaants. * Positive control. Values marked with the same letter in the same column are not statistically different (*p* < 0.05).

**Table 9:** Antiproliferative activity of ungurahui fruits (IC_50_ ± SD, μg/mL).

Sample	MCF-7	Ht-29	HeLa
1	443 ± 57 ^b^	864 ± 121 ^a^	>1000
2	270 ± 15 ^d^	>1000	>1000
3	484 ± 33 ^b^	>1000	>1000
4	685 ± 88 ^a^	723 ± 48 ^b^	>1000
5	366 ± 21 ^c^	765 ± 65 ^b^	>1000
6	255 ± 17 ^d^	>1000	>1000
7	291 ± 25 ^d^	>1000	>1000
8	747 ± 97 ^a^	>1000	>1000
9	>1000	>1000	>1000
Taxol *	13 ± 1 ^e^	15 ± 2 ^c^	11 ± 1

Sample: 1: Bellavista, 2: Hacienda Concepción, 3: Jenaro Herrera, 4: Contamana, 5: Allpahuayo Mishana, 6: San Lorenzo, 7: Atalaya, 8: Palestina, 9: Tunaants. * Positive control. Literals on each column show significant differences among each determination, according to Tukey’s test (*p* < 0.05).

## Data Availability

The data is contained within the article and supplementary materials.

## Supplementary Materials

The following supporting information can be downloaded at: https://www.mdpi.com/article/10.3390/antiox11081598/s1, Figure S1: Palm trees with fruits, Figure S2: ORAC curve with Ungurahui samples, Figure S3: Fatty acid profiles, Figure S4: Example of some UHPLC-Q-Orbitrap spectra, peaks 6, 7, and 16.
