# Supplementary material for: Antioxidant, Antiproliferative and Anti-Enzymatic Capacities, Nutritional Analysis and UHPLC-PDA-MS Characterization of Ungurahui Palm Fruits (Oenocarpus bataua Mart) from the Peruvian Amazon
Source: Antioxidants (Basel). 2022 Aug 18;11(8):1598. doi: 10.3390/antiox11081598 (PMC9404833; doi:10.3390/antiox11081598)
Supplement: Supplementary file 1 [file antioxidants-11-01598-s001.zip › antioxidants-1822489-supplementary.pdf]

Supplementary Materials

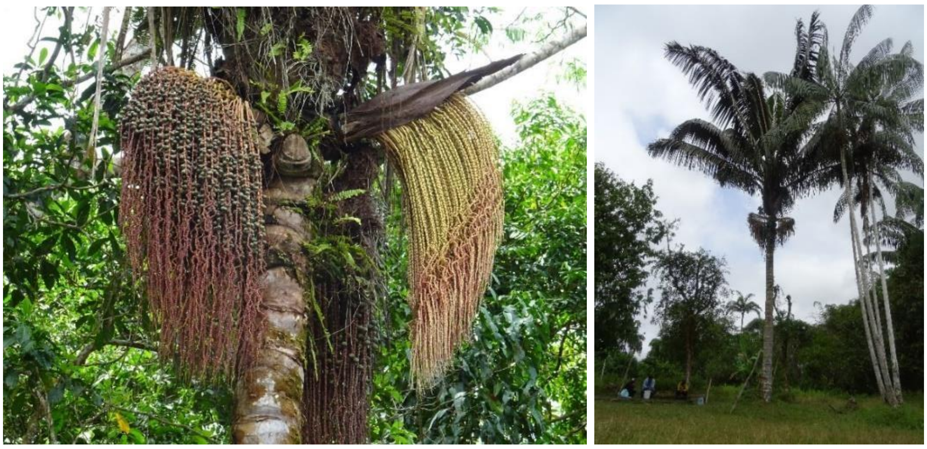

Figure S1. Palm trees with fruits.

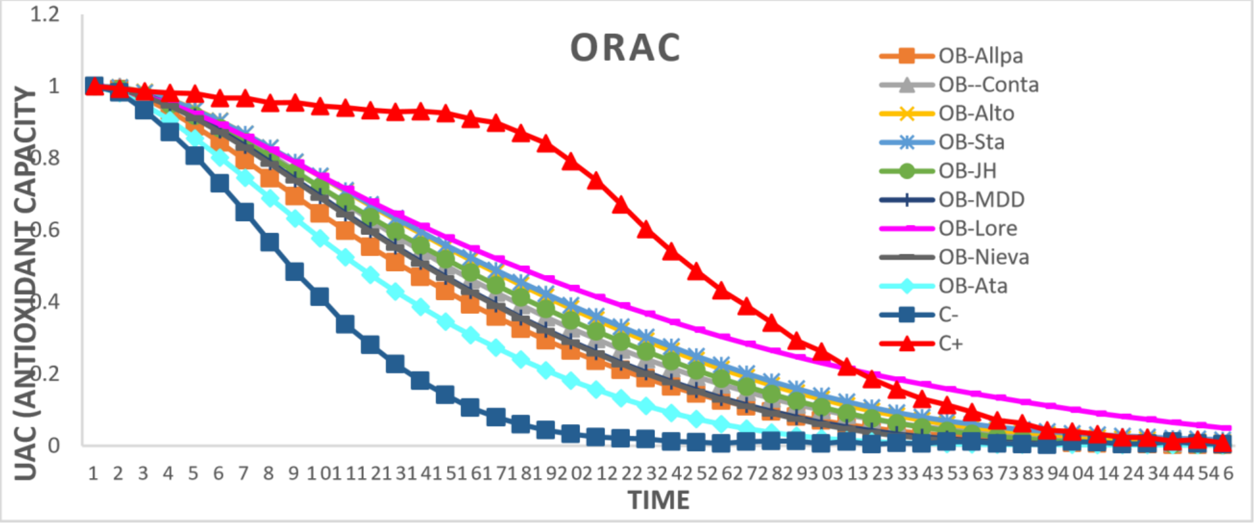

Figure S2. ORAC curve with ungurahui samples.

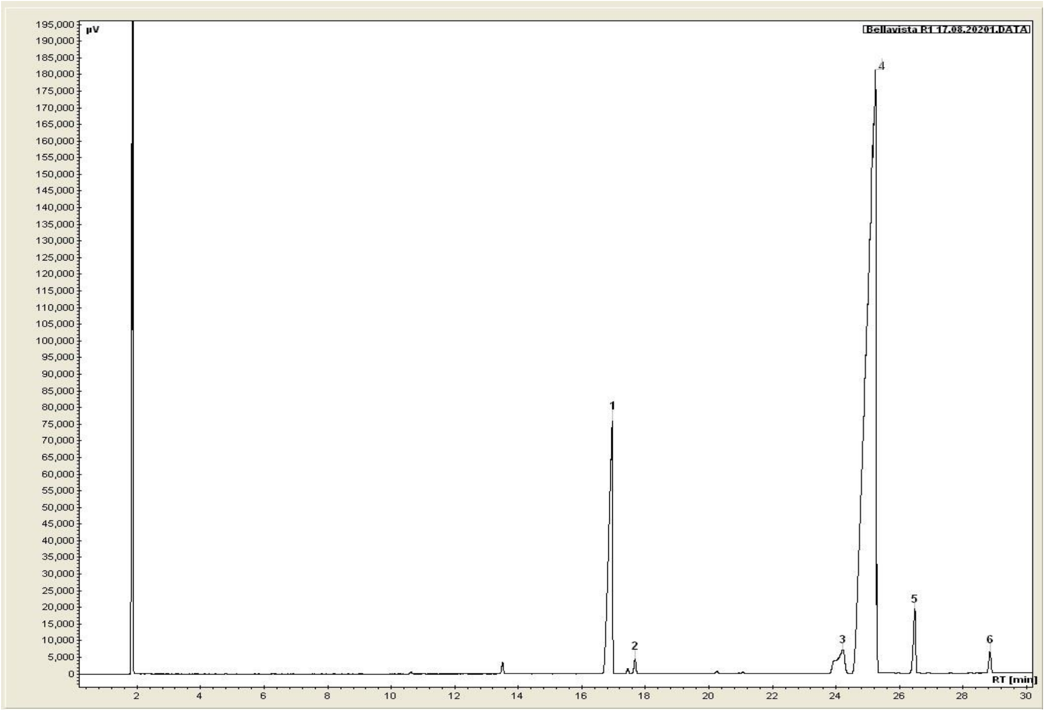

BELLAVISTA

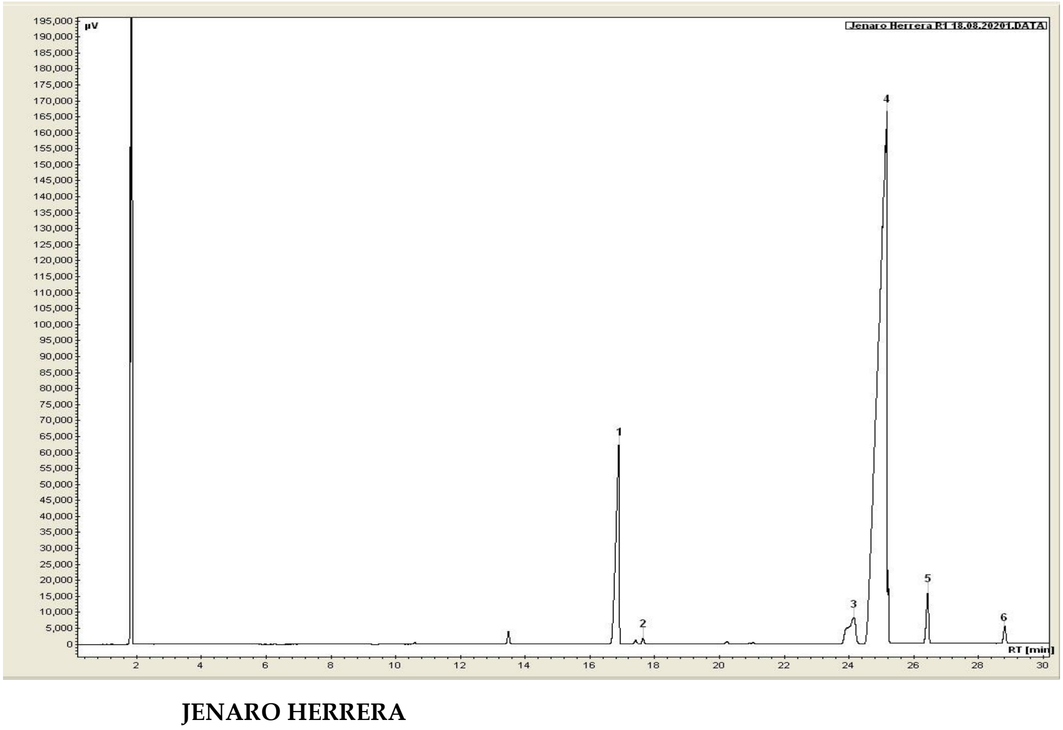

JENARO HERRERA

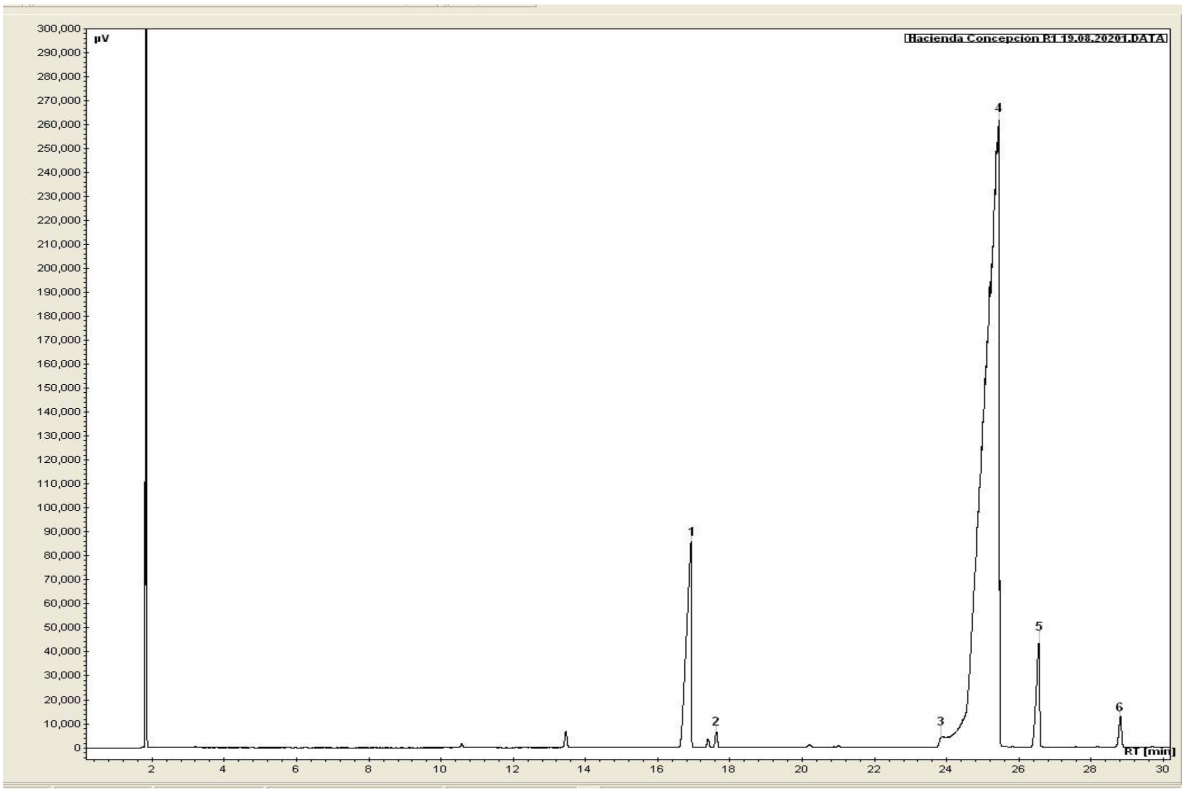

HACIENDA CONCEPCIÓN

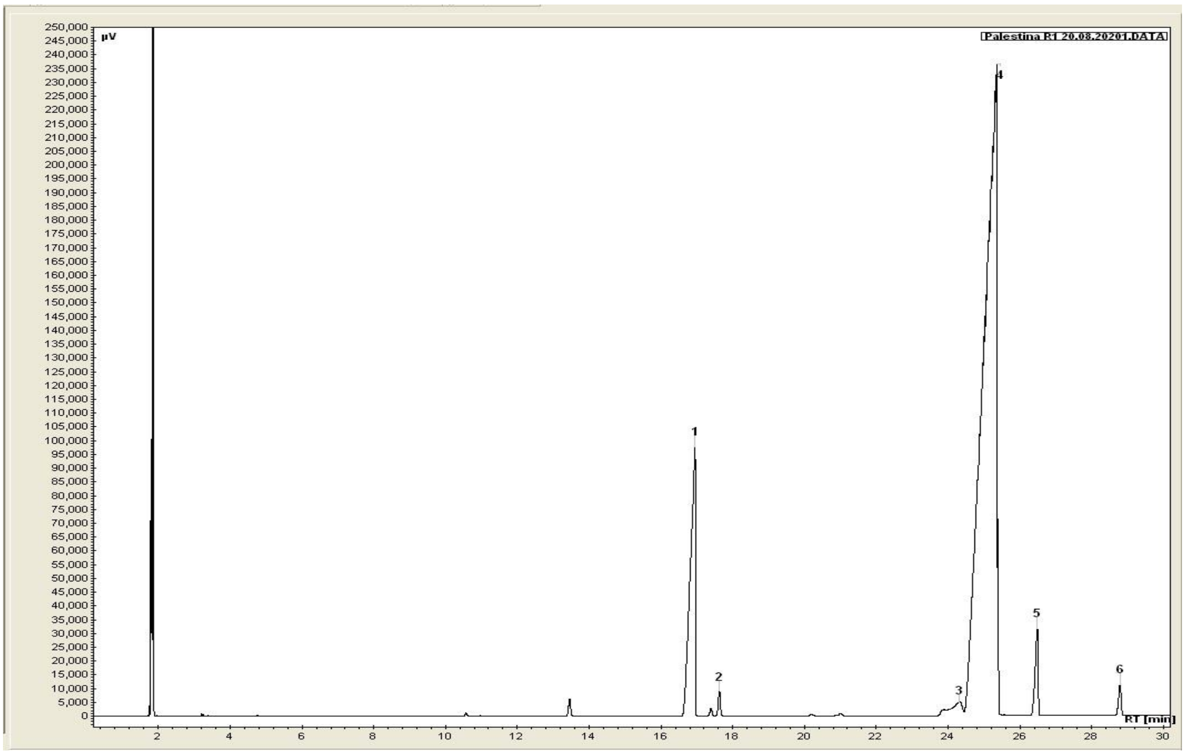

PALESTINA

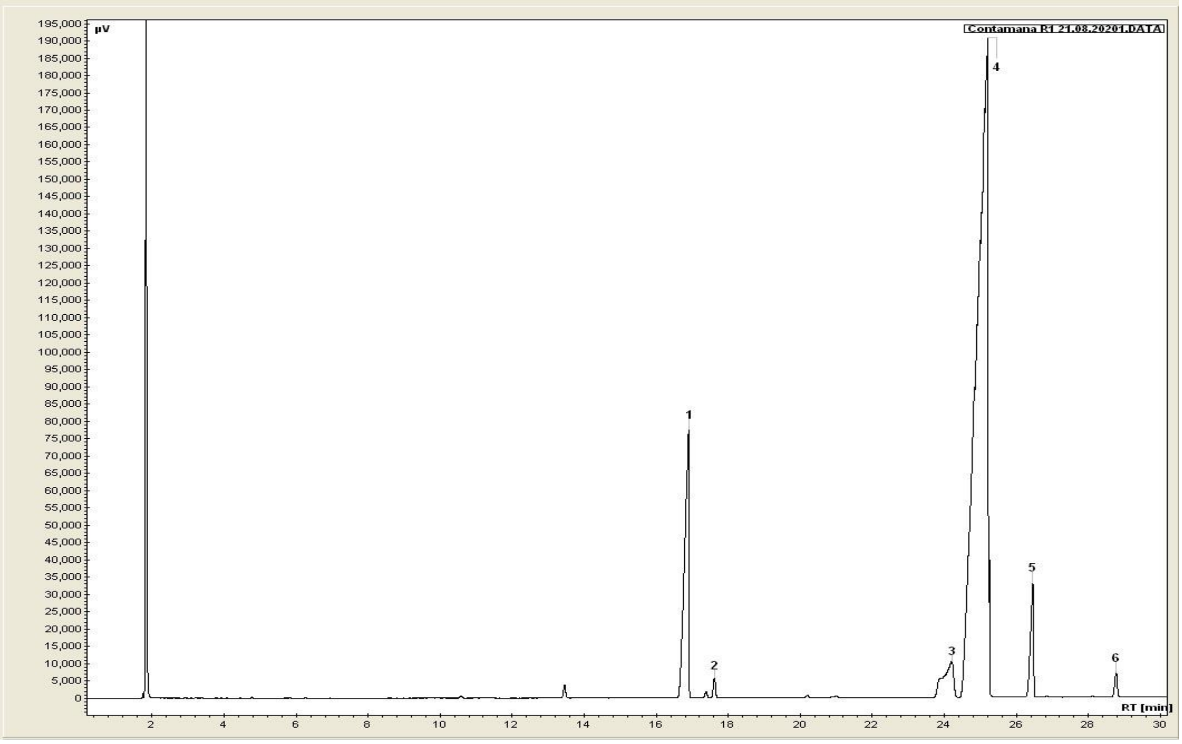

CONTAMANA

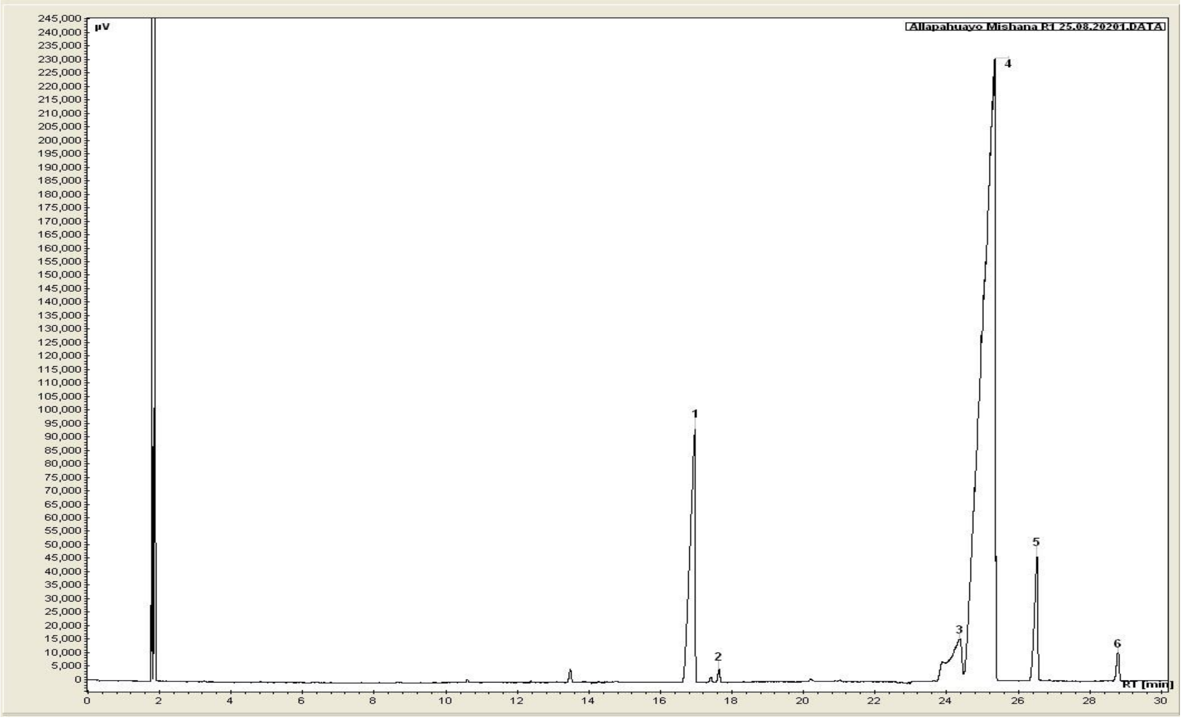

ALLPAHUAYO MISHANA

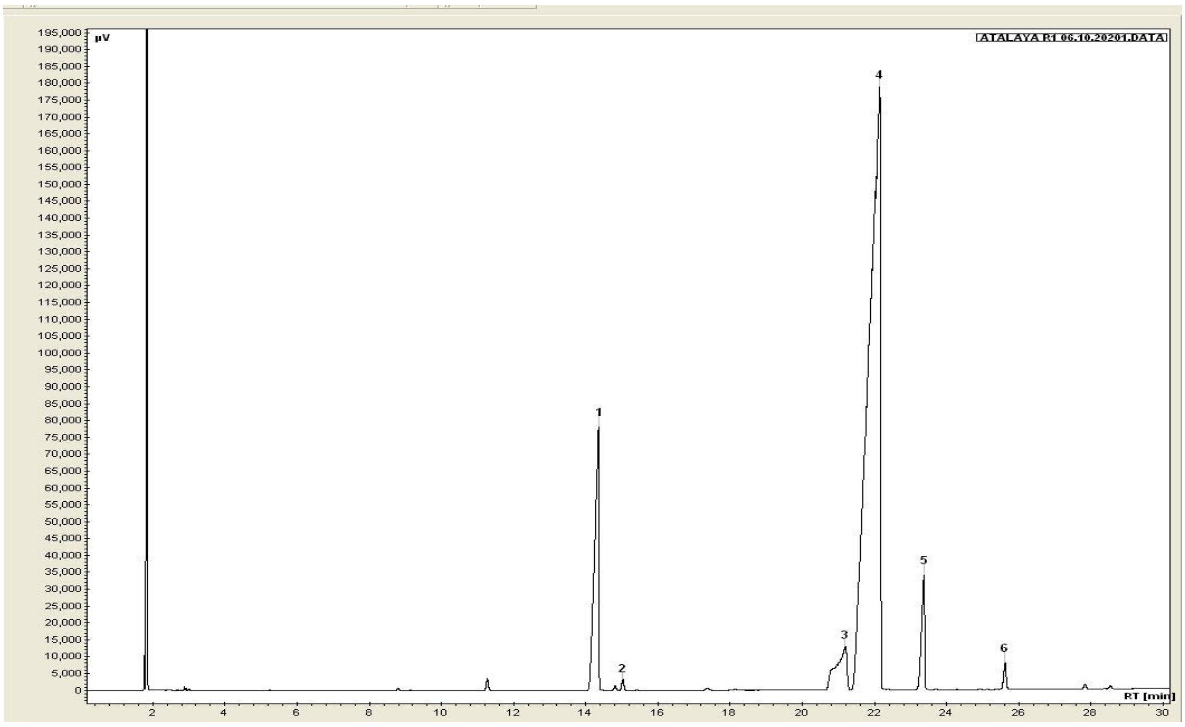

ATALAYA

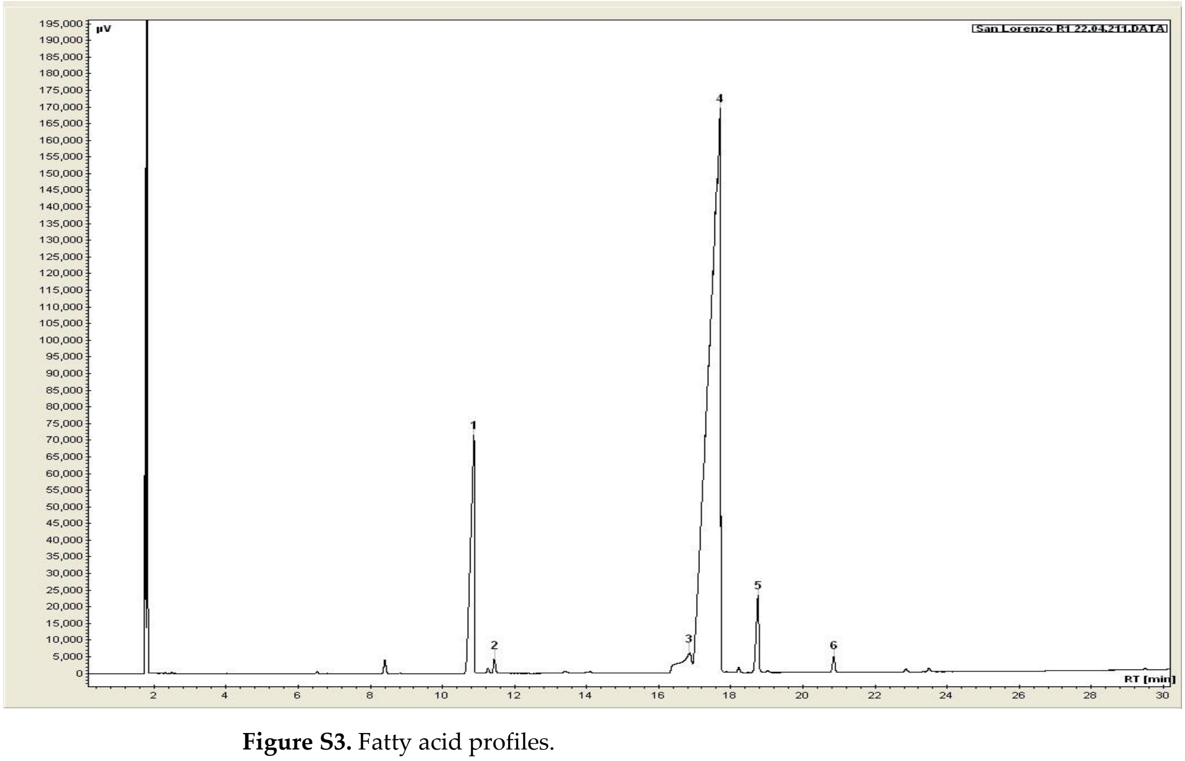

Figure S3. Fatty acid profiles.

Number	Fatty acid
1	Palmitic C16:0
2	Palmitoleic C16:1
3	Stearic C18:0
4.	Oleic C18:1
5	Linoleic C18:2
6	Linolenic C18:3

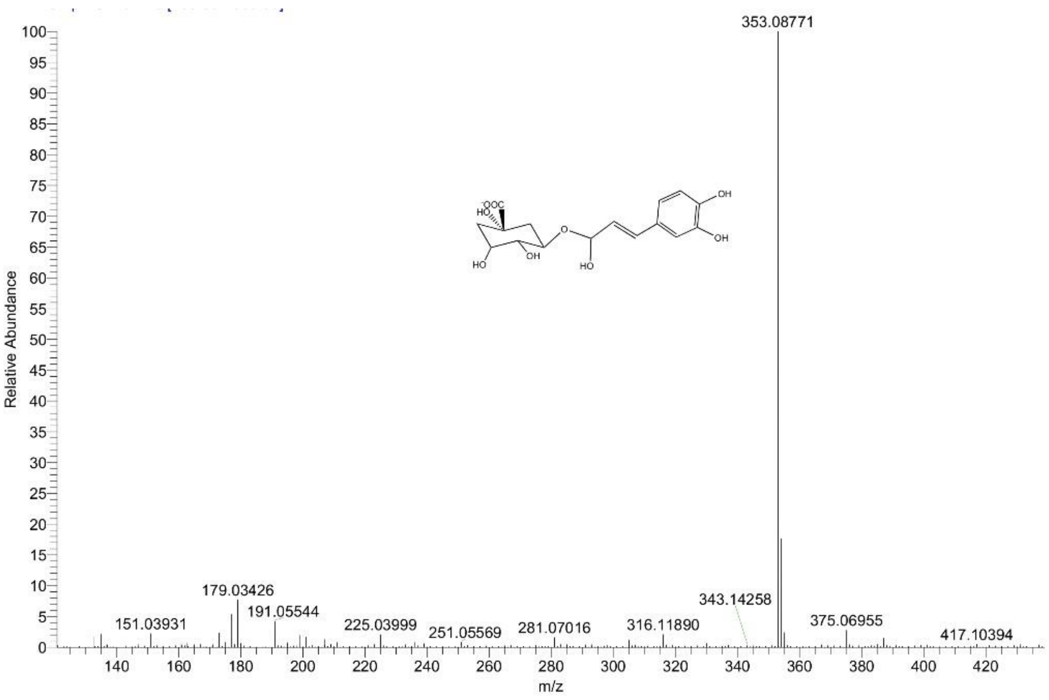
353.08771
179.03426
191.05544
151.03931
225.03999
251.05569
281.07016
316.11890
343.14258
375.06955
417.10394
Relative Abundance
m/z
OH
HO
OOC
O
OH
HO
HO

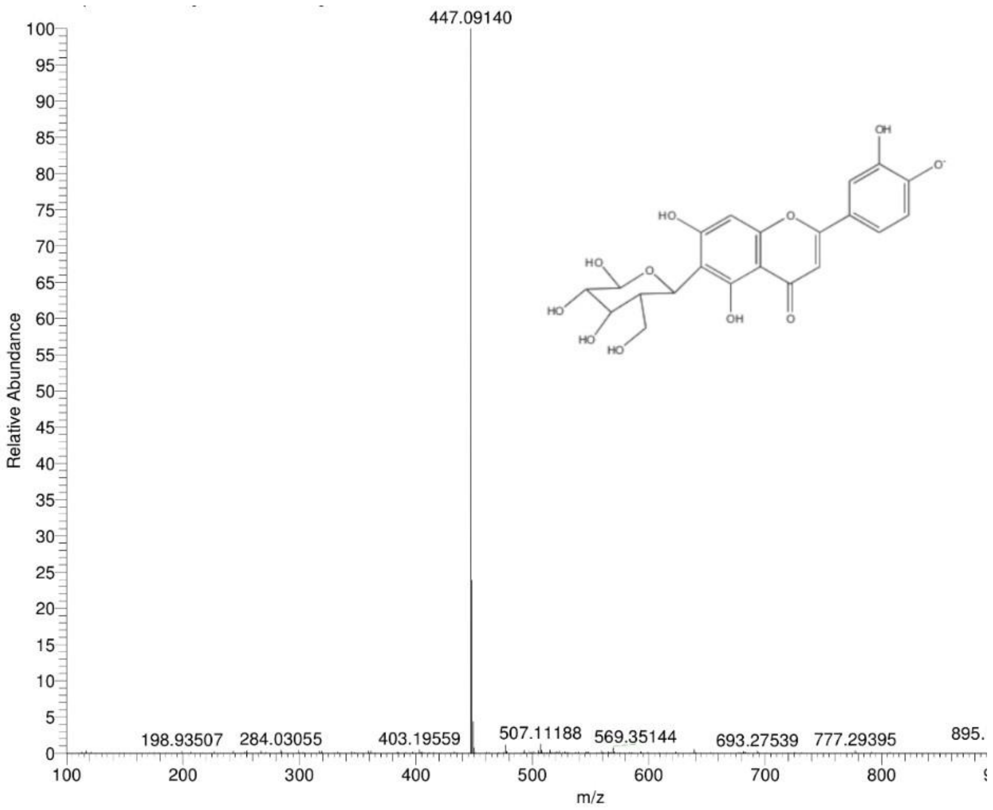
447.09140
198.93507
284.03055
403.19559
507.11188
569.35144
693.27539
777.29395
895.
Relative Abundance
m/z
OH
HO
O
OH
HO
HO
HO
HO
O

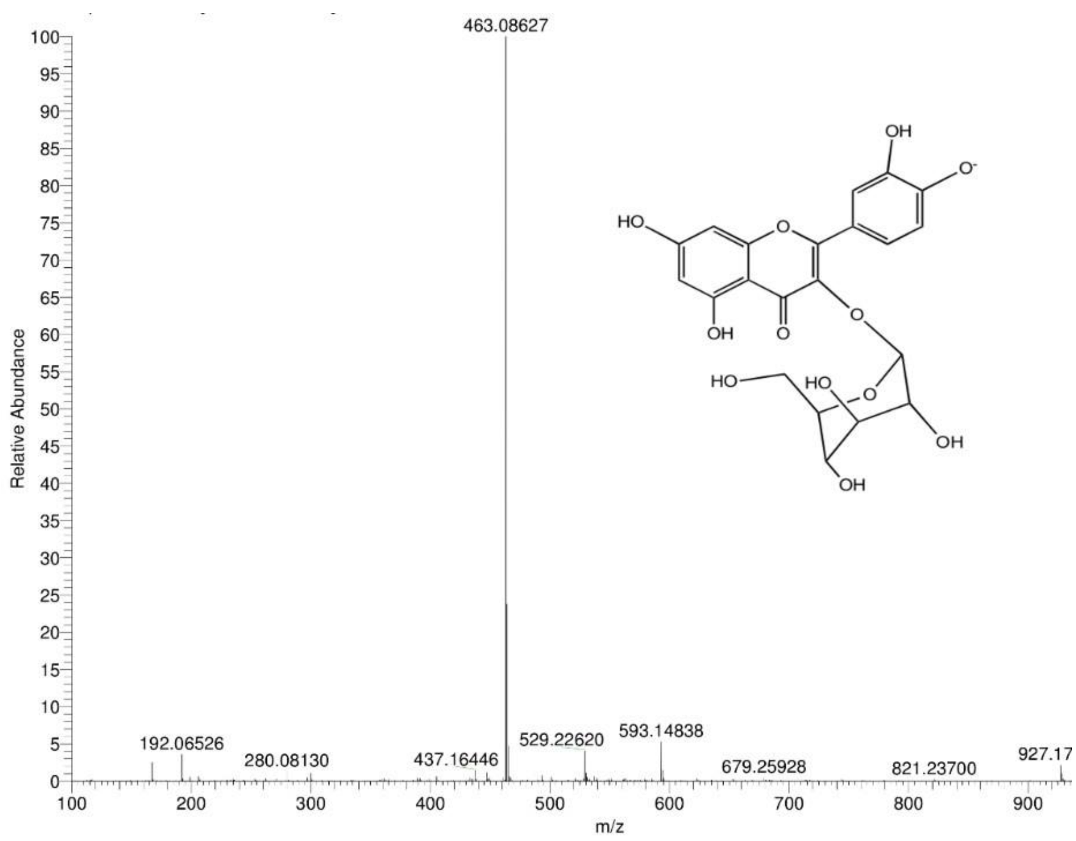

Figure S4. Example Of some UHPLC Q Orbitrap Spectra peaks 6, 7, and 16.
